# Diagnostic performance of artificial intelligence–based imaging and liquid biopsy combined with clinical evaluation for distinguishing benign and malignant pulmonary nodules: a diagnostic network meta-analysis

**DOI:** 10.1097/JS9.0000000000005144

**Published:** 2026-04-28

**Authors:** Yifan Zhao, Guixue Yang, Miao Yang, Xufeng Deng, Zhuoxin Dai, Peng Dai, Kai Wang, Shuangqing Liao, Li Jiang, Jigang Dai, Quanxing Liu

**Affiliations:** a Department of Thoracic Surgery, Xinqiao Hospital, Army (Third Military) Medical University, Chongqing, China; b Hematopoietic Acute Radiation Syndrome Medical and Pharmaceutical Basic Research Innovation Center, Ministry of Education of the People’s Republic of China, Chongqing, China

**Keywords:** artificial intelligence, CT imaging, diagnostic accuracy, liquid biopsy, network meta-analysis, pulmonary nodules

## Abstract

**Background:**

The incidence of pulmonary nodules is rising. In the United States alone, approximately 1.57 million cases are detected annually via low-dose CT screening. This growing number has created an urgent need for accurately differentiating malignant lesions. The current standard methods rely on radiologist interpretation. However, these methods show variable accuracy, with a sensitivity range of 62%–79%. Invasive biopsies, which are often used for further diagnosis, carry complication risks ranging from 15% to 28%. Emerging technologies, such as artificial intelligence (AI) imaging analysis and liquid biopsy platforms, show promising capabilities in pulmonary nodule characterization. Nevertheless, there is a lack of a rigorous methodology for their comparative evaluation.

**Methods:**

We systematically evaluated 20 comparative studies that were published between 2015 and 2024. These studies assessed AI-based imaging methods, which included deep learning architectures and radiomics analysis, as well as liquid biopsy methods, such as ctDNA mutation profiling and methylation-based assays, for the characterization of pulmonary nodules. Bayesian hierarchical modeling was employed to account for the interdependencies among different tests. Evidence certainty was assessed via the GRADE framework.

**Results:**

The CT-Deep approach, when integrated with clinical information, demonstrated the highest sensitivity, with a value of 97.1% and a 90.8%–99.6% credible interval. However, it showed relatively lower specificity, with a value of 67.4% and a 74.4%–92.9% credible interval. Stand-alone liquid biopsy exhibited more balanced operating characteristics, with a sensitivity of 63.1% and a specificity of 82.8%. The hybrid clinical-liquid biopsy strategy showed optimal performance in the summary receiver operating characteristic analysis, with an area under the curve of 0.903. Substantial heterogeneity was observed across studies, with an I^2^ > 50%. Additionally, there were wide confidence intervals for some diagnostic odds ratio estimates, which means that the comparative performance should be interpreted with caution.

**Conclusions:**

AI-enhanced imaging techniques are particularly valuable for high-sensitivity screening applications, where the risk of false negatives is the greatest. On the other hand, liquid biopsy approaches or their combination with clinical assessment may be more suitable for situations that require high specificity. The findings highlight the need for standardized validation protocols and prospective evaluation of combined modality strategies to address the current limitations in pulmonary nodule characterization.

## Introduction

The clinical necessity of accurately differentiating benign from malignant pulmonary nodules is growing increasingly urgent with the widespread implementation of low-dose computed tomography (LDCT) screening programs. These screening efforts identify indeterminate pulmonary nodules in 23%–31% of individuals undergoing screening – an estimated 1.57 million cases each year in the United States alone^[^1,2^]^. Despite the significant clinical burden posed by these findings, current diagnostic approaches continue to present notable limitations. Radiologists’ visual interpretation, while widely used, exhibits inconsistent accuracy, with a reported sensitivity ranging from 62% to 79% and a specificity from 74% to 91%, per multiple meta-analyses^[^3,4^]^. In addition, invasive diagnostic procedures such as biopsy carry considerable risks, including complication rates of 15–28% that encompass complications like pneumothorax and hemorrhage^[^5,6^]^. This diagnostic uncertainty contributes to delayed treatment in approximately 18% of patients with malignant nodules and results in unnecessary invasive procedures in about 33% of those with benign lesions^[^7^]^, thereby underscoring the pressing requirement for more dependable and noninvasive diagnostic methods.

Emerging technological advancements have shown significant promise in addressing these challenges. In particular, artificial intelligence (AI)-driven imaging analysis – especially with convolutional neural networks (CNNs) – has achieved area under the curve (AUC) values of 0.86–0.94 in multicenter validation studies, outperforming radiologists by 12%–17% in sensitivity while maintaining equivalent specificity^[^8–10^]^. For instance, the DeepLN algorithm was found to reduce false positive rates by 29.5% in the National Lung Screening Trial (NLST) dataset, while retaining a sensitivity of 94.1%^[^11^]^. Concurrently, progress in liquid biopsy techniques – particularly those involving methylation-based assays of plasma biomarkers – has shown sensitivity ranging from 76.8% to 83.4% and specificity exceeding 95% in detecting early-stage lung cancer^[^12,13^]^. Notably, the “PulmoSeek” panel, which is based on a 7-gene methylation signature, has shown considerable potential; a prospective study involving 10 000 patients revealed that this panel successfully detected 92% of stage I adenocarcinomas that had been missed by LDCT alone^[^14^]^.

Nevertheless, several critical gaps in the evidence base remain. To date, no studies have directly compared AI-based imaging analysis with liquid biopsy using uniform and consistent reference standards, and existing comparative evaluations are largely confined to single-modality meta-analyses^[^15,16^]^. Moreover, although there is a strong theoretical basis for combining these modalities – leveraging AI’s high spatial resolution in conjunction with the molecular specificity of liquid biopsy – such integrated approaches have not yet been investigated in randomized controlled trials^[^17^]^. Additionally, inconsistencies in validation methodologies, such as differing thresholds for nodule size and variations in follow-up durations, further complicate efforts to compare and evaluate these technologies across studies^[^18,19^]^.

This study seeks to bridge these critical gaps by conducting the first diagnostic network meta-analysis (DNMA) encompassing 20 comparative studies. The analysis employs bivariate hierarchical modeling to appropriately account for the interdependencies among different diagnostic tests – an analytical advancement over conventional pairwise meta-analytic methods^[^20^]^. This study adheres to the PRISMA-DTA (Preferred Reporting Items for Systematic Reviews and Meta-Analyses of Diagnostic Test Accuracy) guidelines^[^21^]^ and employs the revised QUADAS-2 (Quality Assessment of Diagnostic Accuracy Studies 2) criteria to rigorously assess diagnostic test studies^[^22^]^. With this rigorous methodology and comprehensive approach, the study seeks to generate Level Ia evidence to inform and improve clinical decision-making in pulmonary nodule evaluation.

## Methods

### Study design and registration

This diagnostic network meta-analysis (DNMA) follows the PRISMA-DTA guidelines^[^23^]^, adheres to The Cochrane Handbook for Diagnostic Test Accuracy Reviews^[^24^]^, uses the GRADE framework to assess evidence certainty^[^25,26^]^, and builds on prior lung cancer screening methodologies^[^2,7^]^.

HIGHLIGHTS

- First direct comparison of AI-enhanced imaging vs liquid biopsy for pulmonary nodule diagnosis.
- Integrated liquid biopsy (AUC 0.903) outperforms standard imaging; CT deep learning (97.1% sensitivity) and liquid biopsy (82.8% specificity) show strengths.
- Supports clinical integration of liquid biopsy to reduce unnecessary invasive pulmonary nodule procedures.
- Network meta-analysis follows PRISMA-NMA guidelines and uses GRADE for robust evidence synthesis.

Per TITAN Guideline requirements^[^27^]^, we explicitly declare no artificial intelligence tools were used throughout the research process – all work (literature screening, data extraction, statistical analysis, manuscript writing) was completed manually by the team, ensuring compliance with academic transparency standards and traceability.

This systematic review and DNMA also follow the Assessing the Methodological Quality of Systematic Reviews 2 (AMSTAR 2) guidelines^[^28^]^ – a critical appraisal tool for systematic reviews (especially those with meta-analyses). We adhered to AMSTAR 2 criteria, including protocol registration, comprehensive literature search, duplicate study selection/data extraction, risk of bias assessment for included studies, and transparent result reporting, to ensure methodological rigor and reproducibility.

### Eligibility criteria

We included studies involving adults (≥18 years) with pulmonary nodules (4–30 mm) confirmed by histopathology (biopsy/surgery)^[^29,30^]^ or benign cases validated through ≥12-month follow-up^[^3^]^, consistent with British Thoracic Society size thresholds^[^2^]^. Eligible interventions comprised (a) AI-based imaging (deep learning models such as CNNs and transformers^[^31^]^, radiomics analysis^[^32,33^]^, or other machine learning techniques applied to CT/MRI/PET, mirroring technologies from the NLST trial^[^1^]^) and (b) liquid biopsy methods, including ctDNA mutation/methylation profiling^[^34^]^, exosome-based assays^[^35^]^, protein biomarkers (e.g., autoantibodies)^[^36^]^, or multi-omics panels^[^37^]^, analogous to clinically validated tests like PulmoSeek^[^14^]^. Comparators required (1) AI performance benchmarks against radiologist interpretation^[^38^]^, using LUNG-RADS criteria^[^39–41^]^, or (2) concordance testing of liquid biopsy against histopathology^[^42^]^, following CAP/CLIA standards^[^43^]^. Primary outcomes were sensitivity and specificity, with secondary metrics (AUC, PPV/NPV) adhering to ISO 20916:2019 definitions^[^44^]^. Exclusion criteria encompassed nodules >30 mm (due to divergent malignancy profiles^[^18^]^), studies without systematic follow-up or pathology confirmation^[^19^]^, and non-comparative designs^[^15^]^.

### Literature search and screening

#### Search strategy

Databases (PubMed, Embase, Web of Science, Cochrane Library) were searched from January 2015 to December 2024 using terms aligned with prior meta-analyses^[^16,20^]^:

(“artificial intelligence” OR “radiomics”) AND (“liquid biopsy” OR “ctDNA”) AND (“pulmonary nodule” OR “lung cancer screening”)

#### Screening process

Two independent investigators performed duplicate screening of titles and abstracts, and if needed, reviewed full texts of potentially relevant articles to assess study eligibility for inclusion in the analysis^[^45,46^]^. For full-text assessment and any discrepancies, resolution occurred through consensus discussion or adjudication by senior researchers, following PRISMA-DTA protocols^[^23^]^.

### Data extraction and quality assessment

One investigator extracted data from included studies using a standardized worksheet, documenting study identification (first author, publication year, study design), baseline characteristics (sample size, age, sex, smoking history), and outcome data (TP, FP, FN, TN). Detailed data can be found in Supplemental Digital Content Material 3, available at: http://links.lww.com/JS9/H130.

The methodological quality of included studies was assessed with the modified QUADAS-2 tool (Quality Assessment of Diagnostic Accuracy Studies 2) using Review Manager 5 (RevMan 5.4, Cochrane Collaboration, Copenhagen, Denmark)^[^22^]^. This assessment focused on four domains: patient selection, index test, reference standard, and flow/timing, each assessed for risk of bias and applicability concerns relevant to diagnostic accuracy studies.

### Statistical analysis

Statistical analyses were conducted in R 4.3.1 using a Bayesian ANOVA model^[^47^]^ to account for the inherent correlation between sensitivity and specificity, which are jointly determined diagnostic outcomes susceptible to threshold effects^[^20,48^]^. Unlike conventional approaches that independently pool sensitivity and specificity with fixed/random-effects models based on I^2^ statistics, this model directly addresses the bivariate nature of diagnostic accuracy data^[^49^]^. Pooled diagnostic accuracy metrics included summary sensitivity, summary specificity, relative sensitivity, relative specificity, and diagnostic odds ratio (DOR), with hierarchical modeling implemented using the rstan package for Bayesian computation. For comparative evaluation, network meta-analysis was conducted, with results visualized through network evidence graphs (intervention/outcome relationships)^[^50^]^. Summary receiver operating characteristic (SROC) curves were generated to visualize the bivariate relationship between sensitivity and specificity across diagnostic tests, with the area under the SROC curve (AUC) calculated to quantify overall diagnostic performance^[^51–54^]^.

## Results

### Literature screening process and results

**Figure 1.**
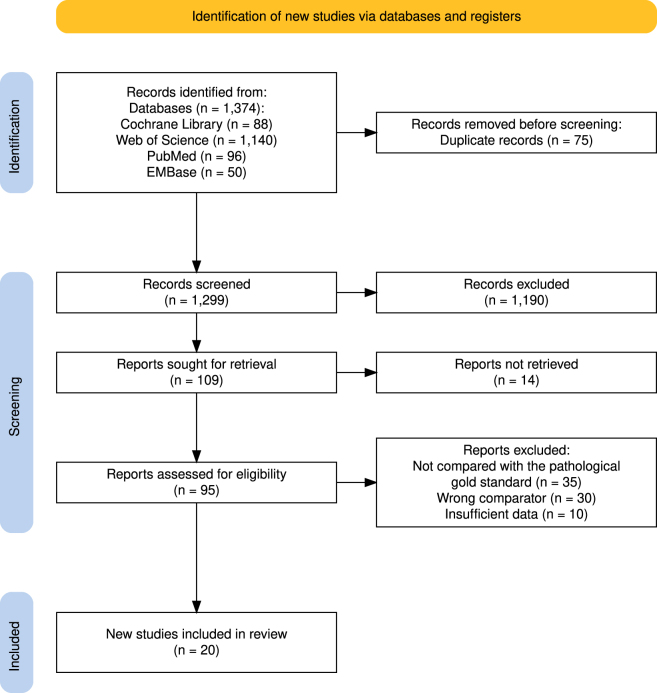
Flowchart of study selection.

A total of 1374 records were initially identified through searches across four electronic databases: the Cochrane Library (n = 88), Web of Science (n = 1,140), PubMed (n = 96), and EMBase (n = 50). After removing 75 duplicate records, 1299 unique records remained for initial screening. During title and abstract screening, 1190 records were excluded, leaving 109 potentially eligible full texts for further assessment. However, full texts could not be retrieved for 14 of these reports. Among the 95 full texts successfully obtained and assessed for eligibility, 35 were excluded for lacking comparison to the pathological gold standard, 30 for using an inappropriate comparator, and 10 for insufficient data reporting. This left 20 studies ultimately included in the final systematic review. The detailed literature screening process is shown in Figure 1.

### Main characteristics of included studies

The included studies encompassed a variety of diagnostic methods and their combinations, including CT-Deep, CT interpreted by radiologists (CT-Radiologist), liquid biopsy, and other diagnostic modalities and their combinations. Basic characteristics of the included studies are summarized in Supplemental Digital Content tables, available at: http://links.lww.com/JS9/H392.

### Study quality

**Figure 2.**
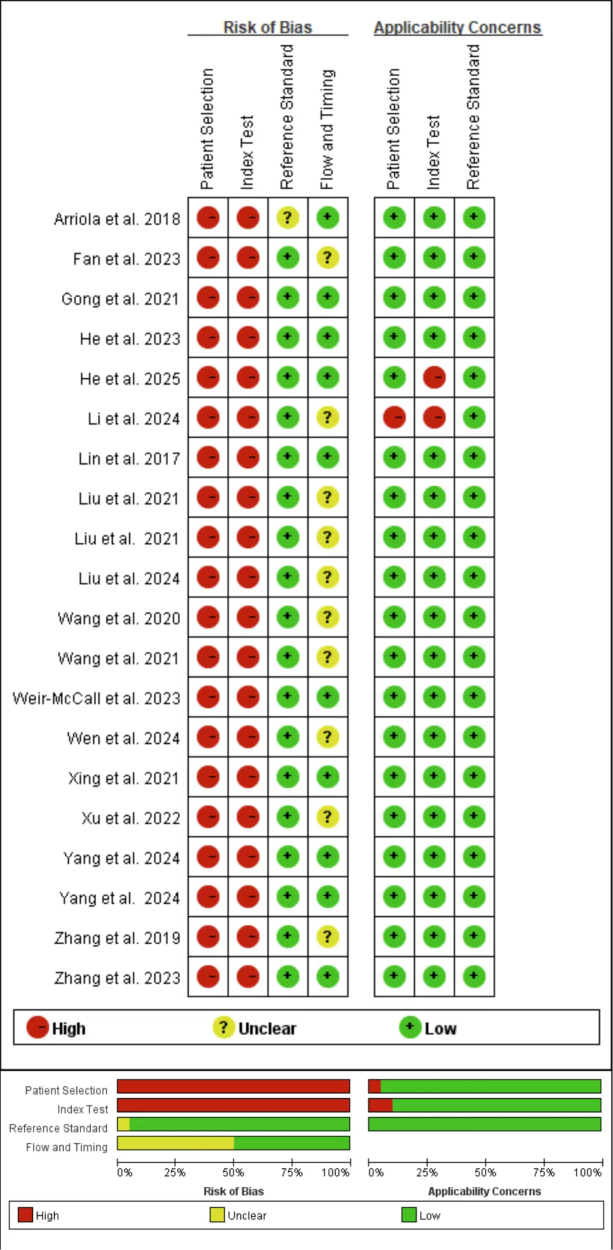
Quality assessment of the studies included using the modified quality assessment of diagnostic accuracy studies.

The methodological quality of the included studies was assessed using the QUADAS-2 (Quality Assessment of Diagnostic Accuracy Studies 2) tool, with results presented in Figure 2. Overall, all studies were judged to be at high risk of bias in the patient selection and index test domains. This pattern indicates potential systematic errors related to how participants were enrolled (e.g., non-consecutive or non-random sampling, exclusion of difficult-to-diagnose cases) and how the diagnostic tests were selected, conducted, or interpreted. Such issues may limit the extent to which the observed accuracy estimates reflect the true diagnostic performance of the evaluated methods.

For the reference standard domain, most studies were rated as having a low risk of bias, although several were judged as “unclear” due to insufficient reporting of how the final diagnosis was established or whether it was applied consistently across participants. The risk of bias for flow and timing was more heterogeneous: while many studies had low risk, some raised concerns because of incomplete follow-up, exclusions from the analyses, or variable intervals between index tests and the reference standard, which may introduce additional uncertainty into the accuracy estimates.

With respect to applicability concerns, patient selection and index test domains were generally rated as low concern, suggesting that the enrolled populations and the evaluated diagnostic strategies were broadly representative of real-world clinical practice for pulmonary nodules. The applicability of the reference standard was also largely appropriate, although a small number of studies had unclear applicability due to limited detail on how the reference diagnosis was determined. Overall, while the clinical scenarios studied appear relevant, the consistently high risk of bias in patient selection and index test domains underscores the need for cautious interpretation of our pooled and comparative findings.

### Network meta-analysis

**Figure 3.**
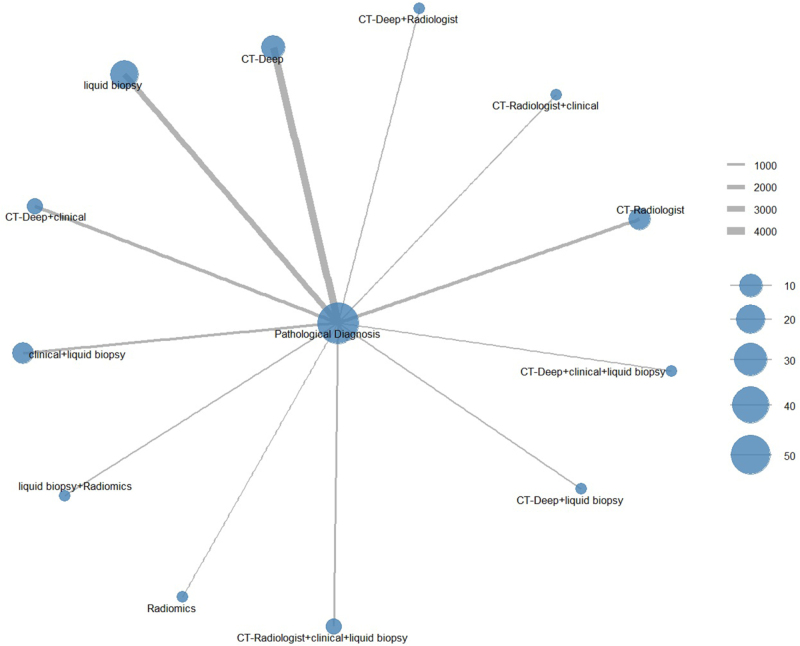
Network of included studies.

A comprehensive network meta-analysis was performed to assess the diagnostic performance of 12 distinct approaches for pulmonary nodule characterization. The network relationships between these diagnostic modalities and the pathological reference standard are illustrated in Figure 3. Bayesian random effects modeling was employed, which revealed significant heterogeneity in the diagnostic capabilities across the various techniques.

**Figure 4.**
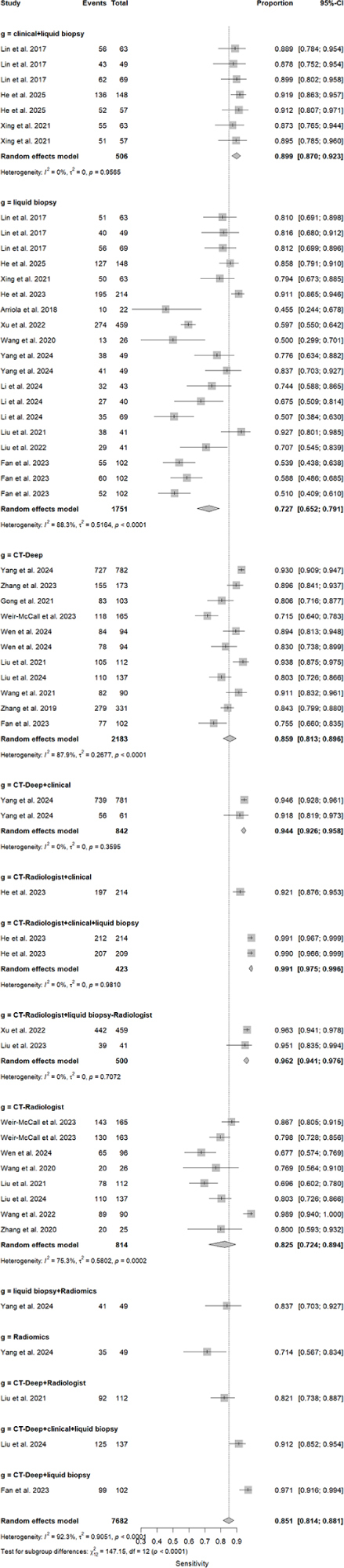
Forest plot demonstrating pooled sensitivity of various diagnostic modalities for detecting lung cancer in pulmonary nodule evaluation. CI, confidence interval.

In terms of sensitivity (Fig. 4), the combination of CT-Deep with clinical assessment demonstrated the highest sensitivity, with a detection rate of 97.1% (95% credible interval: 90.8–99.6). This was followed by the combination of CT-Deep and liquid biopsy, which achieved a sensitivity of 90.0% (75.7–97.2). The clinical-liquid biopsy combination showed moderately high but more balanced sensitivity at 85.2% (74.2–92.9). In contrast, liquid biopsy used alone exhibited the lowest sensitivity among all evaluated methods, at 63.1% (36.1–86.2). Detailed sensitivity statistics for all diagnostic modalities, including Bayesian model metrics, are available in Supplemental Digital Content Material 1, available at: http://links.lww.com/JS9/H128.

**Figure 5.**
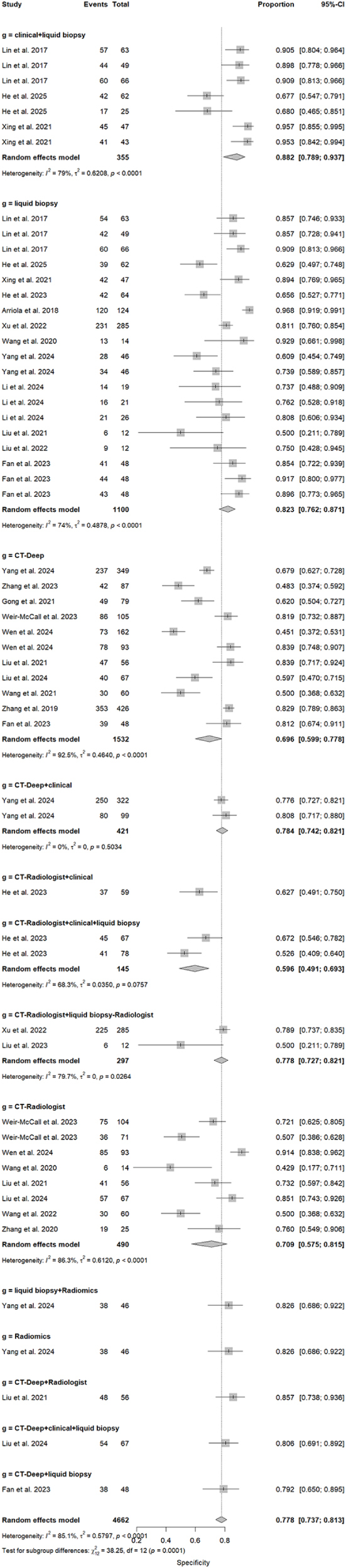
Forest plot illustrating pooled specificity across studies assessing different diagnostic tests for ruling out malignancy in pulmonary nodules.

The specificity results (Fig. 5) displayed a different pattern of performance. Liquid biopsy alone achieved a specificity of 82.8% (79.9–88.3), slightly higher than that of the clinical-liquid biopsy combination at 85.3% (79.9–88.3). Radiomics also showed respectable specificity at 79.3% (73.3–84.3). However, the CT-Deep-based approaches, which exhibited high sensitivity, showed comparatively lower specificity. Specifically, the combination of CT-Deep with clinical assessment had a specificity of 67.4% (74.4–92.9), and CT-Deep combined with liquid biopsy showed a specificity of 73.9% (46.6–92.5). Detailed specificity data (including chain-specific results) can be found in Supplemental Digital Content Material 1, available at: http://links.lww.com/JS9/H128.

**Figure 6.**
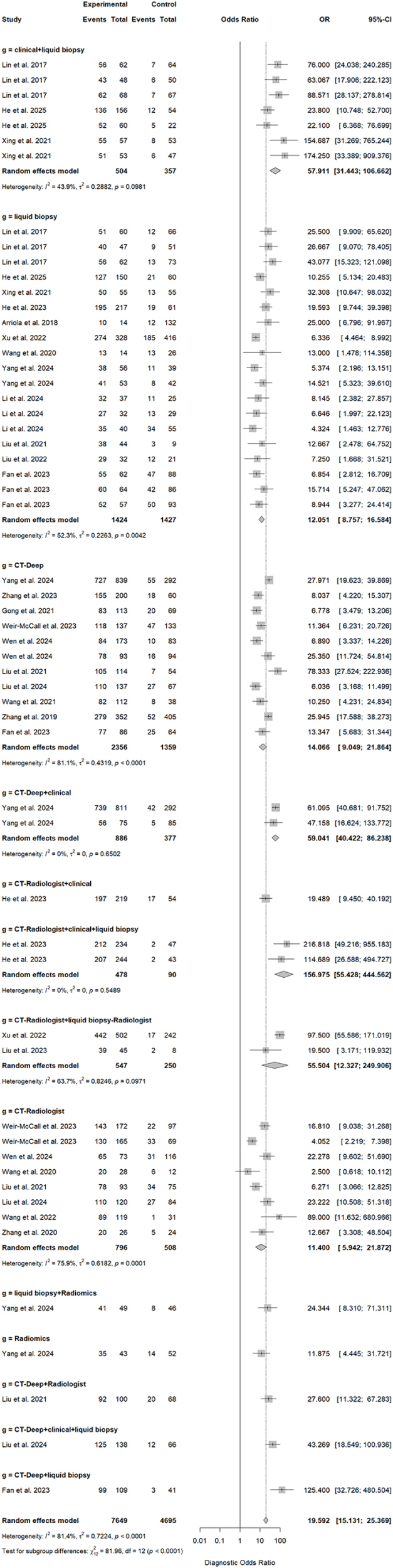
Forest plot showing pooled diagnostic odds ratio of included studies for differentiating malignant pulmonary nodules. DOR, diagnostic odds ratio.

Diagnostic odds ratios (DOR) – which offer a single measure of diagnostic performance combining sensitivity and specificity – showed notable differences among the methods (Fig. 6). The CT-Deep with clinical support approach showed extremely high DOR values (172.78, 13.36–840.40), although the credibility intervals were very wide and should be interpreted with caution. More reliable DOR estimates were obtained for the clinical-liquid biopsy combination (DOR 42.49, 13.05–100.70) and for the integration of CT-Radiologist with clinical-liquid biopsy (DOR 73.48, 18.40–191.85). Stand-alone liquid biopsy showed more modest but precisely estimated performance, with a DOR of 11.11 (7.03–16.97). Comprehensive diagnostic odds ratio (DOR) statistics for all diagnostic approaches are provided in Supplemental Digital Content Material 1, available at: http://links.lww.com/JS9/H128.

The clinical implications of these findings are as follows: while CT-Deep-based methods offer exceptionally high sensitivity, making them particularly valuable for high-risk screening scenarios, their lower specificity and the wide credible intervals around their DORs suggest limitations in generalizability. The clinical-liquid biopsy combination emerged as having the most balanced diagnostic profile, maintaining around 85% sensitivity while also preserving approximately 85% specificity, and thus may represent the optimal approach for routine diagnostic evaluation. Liquid biopsy alone suggests potential advantage utilizing as a secondary or confirmatory test, given its higher specificity but lower sensitivity.

### Subgroup analyses

Subgroup analyses were performed to explore heterogeneity across key variables: validation type (external/internal/none), reference standard (pathology vs mixed follow-up), and methodological approach (deep learning vs radiomics) (Supplemental Digital Content Material 2, available at: http://links.lww.com/JS9/H391).

External validation studies demonstrated significantly higher diagnostic accuracy compared to internal validation and non-validated cohorts. For reference standards, studies using pathology as the gold standard showed comparable accuracy to mixed pathology/follow-up cohorts. Methodologically, radiomics-based approaches yielded higher pooled DOR than deep learning models. The subgroup findings remained consistent with the overall network meta-analysis results, with no significant threshold effect detected in sensitivity analyses (all *P* > 0.05).

These conclusions must be interpreted in light of several limitations. Significant heterogeneity was observed across included studies (*I*^2^ > 50% for most comparisons), likely driven by differences in study protocols, patient populations, and diagnostic workflows. The very wide credible intervals for some DOR estimates – especially those involving combined modalities – indicate substantial uncertainty and underscore the need for cautious interpretation. Additionally, the network meta-analysis included relatively few direct comparisons between certain modality combinations, which increased reliance on indirect evidence.

**Figure 7.**
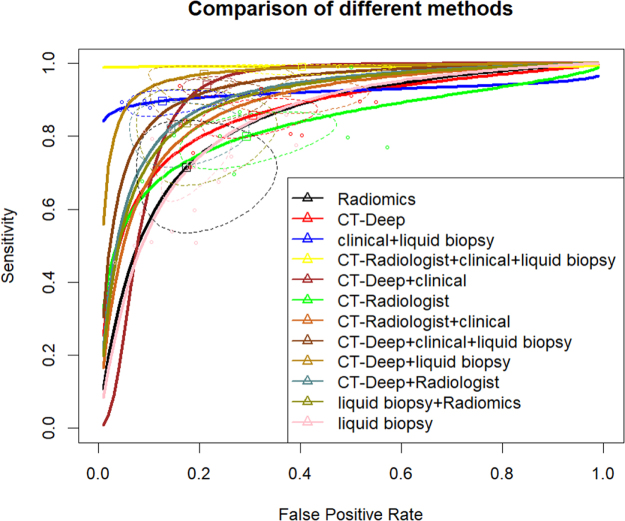
This figure shows the SROC curves of different combinations of diagnostic methods in a network meta-analysis. The horizontal axis represents false positive rate, and the vertical axis represents sensitivity. The diagnostic performance of various combinations of diagnostic methods (such as CT-Deep combined with clinical and liquid biopsy, and radiomics combined with CT, radiologists, liquid biopsy, etc.) was compared.

SROC curve analysis offered further insights into the overall diagnostic performance of the various methods. Figure 7 shows the clinical-liquid biopsy approach had the best SROC performance, with an AUC of 0.903. This indicates that this method achieved the most favorable balance between sensitivity and specificity in the characterization of pulmonary nodules.

## Discussion

This diagnostic network meta-analysis presents what we believe to be the most comprehensive comparative assessment to date of artificial intelligence (AI)-based imaging analysis and liquid biopsy technologies for distinguishing malignant from benign pulmonary nodules. Our findings reveal several key patterns with direct implications for both clinical practice and future research directions in pulmonary nodule management. At the same time, our results also underscore that the current evidence base is constrained by a limited number of primary studies with heterogeneous and often suboptimal methodological quality, particularly in terms of patient selection and reference standards.

The notably high sensitivity of CT-deep learning approaches when integrated with clinical assessment – 97.1%, credible interval [90.8–99.6] – reaffirms and extends earlier single-modality studies, which showed that convolutional neural networks can detect subtle imaging features linked to malignancy. This strong sensitivity suggests that such approaches may be especially valuable in high-risk screening settings, where the consequences of missing early-stage cancers are most severe. However, the relatively low specificity of these methods (67.4%) raises ongoing concerns regarding their potential to generate a high rate of false positives when applied to unselected populations of pulmonary nodules, which could lead to unnecessary invasive diagnostic or therapeutic procedures.

In contrast, liquid biopsy platforms exhibited a qualitatively different performance profile. Stand-alone applications of liquid biopsy showed moderate sensitivity (63.1%) but stronger specificity (82.8%). This pattern aligns with the underlying biological rationale of these technologies: molecular alterations detected in circulating DNA offer high specificity for malignancy, though they may be less sensitive for detecting small or early-stage lesions that have not yet released sufficient quantities of tumor-derived DNA into the bloodstream. The most promising diagnostic performance was observed with strategies that integrated liquid biopsy with clinical assessment. These combined approaches had balanced sensitivity (85.2%) and specificity (85.3%), and the highest SROC-AUC of 0.903. These results indicate that integrated, multimodal diagnostic strategies may provide the most reliable differentiation for the routine evaluation of indeterminate pulmonary nodules. Importantly, these findings support the view that emerging technologies should be used to augment, rather than replace, careful clinical evaluation and radiological judgment in the management of pulmonary nodules.

Our network meta-analytic methodology offered distinct methodological advantages over traditional pairwise meta-analyses by allowing for the simultaneous evaluation of multiple diagnostic strategies while accounting for their interdependencies. This approach revealed important relationships and effects that might otherwise have remained obscured – particularly the enhanced diagnostic performance of combined modality approaches, which have not been adequately assessed in clinical trials to date. To partly address between-study heterogeneity and explore the robustness of our findings, we performed prespecified subgroup analyses stratified by reference standard (histopathology versus clinical/radiological follow-up), study design (prospective versus retrospective), AI-based imaging modality (deep learning–based versus conventional radiological/radiomics-based approaches), and validation strategy (external validation versus no external validation. These analyses generally supported the main patterns observed in the overall network, although the statistical power within some subgroups remained limited.

Nevertheless, we also identified several significant limitations within the existing body of evidence. These include considerable heterogeneity across studies (I^2^ > 50%) and unexpectedly wide credible intervals for certain diagnostic odds ratio estimates. Such variability likely stems from technical factors – such as differences in AI model architectures, molecular targets for liquid biopsy, and imaging protocols – and differences in study populations, nodule selection criteria, and diagnostic reference standards. In particular, many studies enrolled highly selected patient populations and used non-uniform or incompletely reported reference standards, which contributed to a high risk of bias in patient selection and index test interpretation in our quality assessment. Moreover, key methodological details – such as AI model training and validation procedures, liquid biopsy assay targets, and nodule characterization metrics – were frequently under-reported, further complicating cross-study comparisons. Future primary diagnostic studies should therefore adopt more standardized and transparent designs, including clearly defined eligibility criteria, uniformly validated reference standards (for example, centralized pathology review or histopathological confirmation combined with at least 12-month follow-up where appropriate), and comprehensive reporting of critical methodological parameters for both imaging and liquid biopsy components.

From a clinical implementation standpoint, our findings point to distinct and context-specific roles for these technologies. For high-risk screening scenarios where maximizing sensitivity is critical, CT-deep learning approaches appear to be well justified, despite their limitations in specificity. Conversely, when evaluating incidentally detected indeterminate nodules, the more balanced sensitivity and specificity of integrated liquid biopsy strategies may be preferable, as they help reduce unnecessary invasive procedures. The moderate sensitivity of liquid biopsy when used alone suggests that it is currently best employed as a complementary tool rather than a replacement for imaging-based assessment.

Looking ahead, we identify three key priorities for advancing this field. First, the establishment and adoption of standardized evaluation protocols across both imaging and liquid biopsy platforms will be essential to facilitate reliable comparisons and support broader clinical translation. Second, prospective validation of combined modality algorithms in clinically representative populations remains a critical unmet need. Finally, formal cost-effectiveness analyses should accompany evaluations of technical performance to better understand the economic implications and feasibility of implementing these technologies across diverse healthcare systems with varying resources. In addition, as suggested by recent translational research, the integration of advanced imaging features with multi-omics data (including genomic, proteomic, and metabolomic profiles) holds substantial promise for further optimizing joint diagnostic models. However, the current literature contains very few studies that combine AI-enhanced imaging, liquid biopsy, and multi-omics data in a manner amenable to quantitative synthesis; this represents an important avenue for future investigation once a sufficiently robust and standardized evidence base becomes available.

While this analysis has key strengths – including a comprehensive literature search, rigorous methodological approach, and alignment with relevant clinical context – we acknowledge limitations. The relative paucity of direct, head-to-head comparisons between diagnostic modalities necessitated a greater reliance on indirect evidence within our network meta-analysis. Additionally, variability in study quality and the potential for publication bias may have influenced the reliability of certain comparisons. Moreover, we were unable to fully explore how diagnostic performance varies across important clinical subgroups – such as differences in nodule size or patient risk profiles – due to inconsistent reporting across included studies. In particular, granular data on nodule size distributions and patient-level risk factors were often lacking or heterogeneously reported, precluding robust network meta-analyses stratified by these clinically relevant variables. Future studies should systematically collect and report such detailed subgroup information to enable more nuanced assessments of test performance across different risk strata.

In conclusion, this network meta-analysis demonstrates that AI-enhanced imaging and liquid biopsy technologies each offer unique and complementary strengths for the evaluation of pulmonary nodules. Their strategic integration, tailored to specific clinical contexts and nodule characteristics, appears to represent the most promising path forward. These findings provide Level Ia evidence that can inform the development of clinical practice guidelines^[^55^]^ and should help guide the evolving research agenda in this rapidly advancing field.

## Data Availability

The data supporting the findings of this study are available from the corresponding author upon reasonable request. The datasets used and/or analyzed during the current study are derived from publicly available sources as cited in the manuscript or were generated during the study.

## Research registration unique identifying number (UIN)

PROSPERO. CRD420251035117. https://www.crd.york.ac.uk/PROSPERO/view/CRD420251035117
